# Resilience mediates the effect of peer victimization on quality of life in Chongqing adolescents: from a perspective of positive childhood experiences

**DOI:** 10.3389/fpsyg.2023.1186984

**Published:** 2023-07-26

**Authors:** Liya Deng, Yang Liu, Hong Wang, Junjie Yu, Liping Liao

**Affiliations:** ^1^Department of Maternal and Child Health and Adolescent Health, School of Public Health, Chongqing Medical University, Chongqing, China; ^2^Research Center for Medicine and Social Development, School of Public Health, Chongqing Medical University, Chongqing, China

**Keywords:** peer victimization, quality of life, resilience, positive childhood experiences, adolescents

## Abstract

**Background:**

Peer victimization is a harmful experience that contributed to one's psychological problems, physical health deterioration, and so on. Quality of life (QoL) is an important indicator of adolescent health assessment. To identify potential pathways of positive experiences in preventing peer victimization's detrimental effects and then provide intervention ideas for adolescent health, this study was conducted to examine the relationship between peer victimization and QoL in Chongqing adolescents and discover whether resilience plays a mediating role and positive childhood experiences (PCEs) act as a moderating role in the relationship.

**Methods:**

Data were the first follow-up of a cohort study conducted in four complete middle schools in two districts of Chongqing, China. Self-designed peer victimization items, the Connor–Davidson Resilience Scale, the Adolescent Quality of Life Scale, and the Benevolent Childhood Experiences Scale were used. We investigated the differences and correlations in peer victimization, QoL, and resilience between the two PCEs groups. Mplus version 8.3 was used to analyze the mediating role of resilience and the moderating role of PCEs in peer victimization and QoL.

**Results:**

Peer victimization, resilience, and QoL differed between the two PCEs groups (*P* < 0.001). Peer victimization negatively correlated with QoL and resilience, while resilience positively correlated with QoL (*P* < 0.001). In the models with total QOL as the dependent variable, the indirect effect was −0.431 (8.08% of the total effect) in the low-PCEs group vs. −2.077 (41.97% of the total effect) in the high-PCEs group. In the models with four dimensions of QOL as the dependent variable, the indirect effects ranged from −0.054 to −0.180 (6.07–12.95% of the total effects) in the low-PCEs group and from 0.295 to −0.823 in the high-PCEs group (35.89–68.76% of the total effects). Both total and indirect effects were significant (*P* < 0.05). In addition, the differences in indirect effects were significant between the two PCEs groups (*P* < 0.05), while differences in total and direct effects were almost not apparent.

**Conclusion:**

Resilience partially mediated the effect of peer victimization on QoL in Chongqing adolescents, and PCEs moderated this mediation. Schools, families, and society should focus on resilience intervention and prioritize the enhancement of PCEs for improving adolescent QoL.

## 1. Introduction

Adolescence is an important transitional stage in life. Interactions with the social environment shape an individual's capabilities to enter adult life smoothly at this stage. Individuals also gain physical, cognitive, emotional, social, and economic resources from this period to provide the foundation and benefits for their present and future and the wellbeing of the next generation (Patton et al., 2016). Compared to adults, adolescents are more sensitive to the social environment and more vulnerable to external factors (Blakemore and Mills, 2014). Infectious diseases, malnutrition, poor sexual behaviors, injuries, violence, unhealthy lifestyles, decreased family stability, environmental degradation, etc. can pose significant threats to adolescent health and wellbeing (Patton et al., 2016). Therefore, investing in adolescent health has proven to be an important strategy for promoting human health (Patton et al., 2016). To promote adolescent health, it is necessary to further understand their subjective perceptions (Mikkelsen et al., 2022). Quality of life (QoL) is defined by the World Health Organization (WHO) as the subjective experience of individuals in different cultures and value systems about their goals, expectations, standards, and concerns related to their living conditions (Malibary et al., 2019). It usually includes the four dimensions of physical health, mental health, social relationships, and environmental conditions (Malibary et al., 2019). QoL is currently considered an indicator that well reflects the status and quality of one's life activities and has been used in several studies. For example, QoL has been used as an assessment of health status in the treatment of cancer patients (Trask et al., 2009), patients with critical illnesses (Hofhuis et al., 2009), and patients with diabetes mellitus (Hogg et al., 2012). Numerous studies have also used QoL to assess the health effects of polycystic ovary syndrome (Kaczmarek et al., 2016), type 1 diabetes (Cruz et al., 2018), eating disorders (Wu et al., 2019), and COVID-19 (Nobari et al., 2021) on adolescent health. Therefore, focusing on the QoL of adolescents can help us understand their health more conveniently.

According to the trauma theory (Herman, 1992), most adverse childhood experiences (ACEs) are traumatic because they will harm people's physical and psychological health (Chen et al., 2021). As a type of ACEs (Almuneef et al., 2018; Gette et al., 2022), peer victimization involves harmful acts of aggression intentionally committed by the bully, which may occur between individuals and groups in a context of power imbalance (Marsh et al., 2023). The three main dimensions of peer victimization include verbal (e.g., being called insulting nicknames, hurtful joking, and teasing), physical (e.g., being hit, being thrown with things, and being physically threatened), and social/relational (e.g., isolation and exclusion from activities by peers) (Marengo et al., 2019; Marsh et al., 2023). The occurrence of peer victimization is also widespread worldwide. For example, a meta-analysis including 80 studies showed the prevalence of peer victimization to be approximately 36% (Modecki et al., 2014), and this prevalence among school-age children in Western countries could range from 9 to 25% (Menesini and Salmivalli, 2017). In China, a review indicated that the prevalence of peer victimization ranged from 2 to 66% in mainland China, 24 to 50% in Taiwan, and 20 to 62% in Hong Kong (Chan and Wong, 2015). A cross-sectional study in seven Chinese provinces showed that the prevalence of peer victimization in schools reached 26.1%, and witnessing bullying by others also reached 28.9% (Han et al., 2017).

Peer victimization has been shown to be a critical risk factor for adolescent development. Two reviews of longitudinal studies have shown that peer victimization has far-reaching negative effects on adolescent victims, such as psychological problems, deterioration of physical health, maladaptive behaviors, and impaired social relationships (Reijntjes et al., 2010; Turanovic, 2022). For adolescents, having a positive self-image, healthy friendships, and strong familial ties are apparently important for their QoL. When these aspects are severely impacted (e.g., the presence of physical and psychological problems, a lack of self-confidence, and impaired social relationships), their QoL will be threatened (Helseth and Misvaer, 2010). A few observational studies lent credence to this opinion. In Chinese adolescents, peer victimization was significantly associated with poorer health-related QoL (Chen et al., 2018). One longitudinal study in Dutch adolescents found that moderate and high peer victimization resulted in lower levels of future life satisfaction (Sumter et al., 2012). Another longitudinal study suggested that experiences of peer victimization at the age of 10–11 years negatively predicted QoL at the age of 12–13 years (Forbes et al., 2019). In addition, a prospective intervention study showed that reductions in peer victimization improved subsequent QoL, while QoL continued to decline if peer victimization continued to develop (Jantzer et al., 2022). Thus, promoting QoL in adolescents requires attention and attempts to buffer the negative effects of peer victimization.

Resilience is considered to be an individual's ability to positively adapt to the environment, which helps individuals successfully adapt to adversity and promote positive development (Chen et al., 2021). According to the compensatory model of resiliency and the protective factors model, resilience not only acts as an independent positive factor influencing health outcomes but also moderates the relationship between traumatic experiences and health outcomes (Crandall et al., 2019). The relationship between resilience and QoL in adolescents has long been studied, and the results have shown that resilience has a positive association with QoL. For example, Martín-Pérez et al. found that high levels of resilience were associated with better psychological dimensions of QoL in Spanish adolescents (Martín-Pérez et al., 2022). Maheri et al. (2019) and Tang et al. (2022) also found the same positive association in Iranian and Chinese adolescents, respectively. Three intervention studies showed that resilience was an important factor in improving QoL in adolescents with complex congenital heart disease, cancer, and diabetes (Lee et al., 2017; Steineck et al., 2019; Wu et al., 2023).

As a psychological trait, resilience develops through the continuous interaction between the environment and the individual. As shown by the resiliency theory, multiple systems (e.g., individuals, families, neighborhoods, and schools) interact to affect the course of development, and resilience itself is constantly evolving within individuals and systems (Crandall et al., 2019). Given the characteristic of resilience, it is possible that negative environmental factors such as peer victimization could contribute to diminished resilience. For example, Ran et al. discovered that peer victimization caused adolescents to have self-harming behaviors by diminishing resilience (Ran et al., 2020). Anderson et al. (2022) found that peer victimization could influence adolescent depression and anxiety symptoms by affecting resilience. Thus, the above results suggested that resilience could act as a mediating variable between peer victimization and health outcomes. However, resilience has only been thought of as a moderating factor between peer victimization and QoL in some observational studies. For example, Víllora et al. (2020) found that resilience moderated the relationship between poly-bullying victimization and subjective wellbeing although this effect was weak. Martín-Pérez et al. (2022) also found that resilience moderated the relationship between physical and verbal peer victimization with “mood and emotions” dimensions of QoL. Based on the above evidence, it is necessary to explore the mediating role of resilience on the negative effects of peer victimization.

Positive childhood experiences (PCEs) refer to the individual's positive experiences of perceived internal and external safety, security, and support, and positive and predictable QoL before the age of 18 years (Narayan et al., 2018). The Positive Youth Development Framework suggests that a supportive surrounding environment contributes to the adaptive development of adolescents and helps prevent the occurrence of negative outcomes (Lerner et al., 2009). Existing research studies showed positive interpersonal supports (e.g., parental warmth and teacher or peer support) may reduce the risk of peer victimization (Natvig et al., 2001) and its negative effects such as depression (Yin et al., 2017). It was also proven that better school safety often meant less peer victimization (Blosnich and Bossarte, 2011; Díaz et al., 2021). The above studies have shown the debilitating effect of PCEs on peer victimization.

When it comes to the association between PCEs and QoL in adolescents, previous studies have found higher social support has not only been associated with better QoL (Herzer et al., 2011; Singstad et al., 2021) but also played a moderating (Lo et al., 2021) and mediating (Tang et al., 2022) role in the impact of some negative experiences on QoL. Moreover, there were many studies that explored the relationship between PCEs and resilience. Marçal and Maguire-Jack (2022) discovered that neighborhood support could serve as a powerful resilience resource for teens. A review of adolescents of transgender and gender nonconforming (TGNC) found that good relationships with family members (especially with parents), social support except for parents, and better school safety or inclusive school climate promoted their resilience (Tankersley et al., 2021). Even in some cases, divine support (support from God or a higher power) can help adolescents increase resilience (Gower et al., 2020). Based on these results, there was an effect of PCEs on peer victimization, resilience, and QoL. However, no studies have examined the effects of PCEs on the relationship among peer victimization, resilience, and QoL using a specific positive childhood experience scale.

Previous studies have examined the relationship between peer victimization and QoL. However, whether resilience acts as a mediator between the two remains unclear. Moreover, PCEs were related to the three variables above, but no study has discussed how PCEs affect the mediation mechanism of resilience in peer victimization and QoL. We hope to identify potential pathways of positive experiences in preventing peer victimization's detrimental effects and then provide intervention ideas for the physical and mental health of adolescents by exploring the mediating role of resilience and the moderating role of PCEs between peer victimization and QoL. To achieve these goals, the present study utilized a sample of adolescents from Chongqing, China, for investigation. We hypothesized that (1) scores of QoL/dimensions of QoL, peer victimization, and resilience will be different across two levels of PCEs; (2) QoL/dimensions of QoL, peer victimization, and resilience will be correlated across two levels of PCEs; and (3) resilience will show a mediating effect in the association between peer victimization and QoL/dimensions of QoL, and the path coefficients will be different in the high-PCEs group vs. the low-PCEs group.

## 2. Materials and methods

### 2.1. Sample and procedure

The cross-sectional data were the first follow-up part in May 2022 of the Adolescent Behavior and Health Survey Cohort Study conducted in four complete middle schools (the schools include middle and high school students) in two districts of Chongqing. According to the administrative and economic development level of Chongqing, it is divided into central urban areas and districts. Then, Yubei District and Qijiang District were selected in two areas, and two schools were chosen for each region according to the principle of convenient sampling. Since the study lasted for 2 years, the loss of follow-up due to further education needed to be considered. Therefore, we chose students in 7th and 10th grade to follow. According to the use of a random number table, four to five classes in each grade were chosen for the survey. The inclusion criteria were that students could independently understand and complete the questionnaire.

The sample size for the first follow-up section was 1,747 (including 7th and 10th graders). The participants were investigated with a self-reported questionnaire, which mainly included scales for general demographic variables, peer victimization, resilience, QoL, and PCEs. Cases with missing demographic variables (unable to supplement, 6 individuals), complete absence of one or more instruments (28 individuals), and apparent logical anomalies in variable values (7 individuals) were first eliminated to obtain a sample of 1,706. The retention rate was 97.71%.

### 2.2. Instruments

#### 2.2.1. General demographic variables

The general demographic variables were collected using self-made items: school location (Qijiang/Yubei), gender (male/female), grade (grade 7th/10th), family residence (countryside/township/urban), whether single child (yes/no), and whether living in school (yes/no).

#### 2.2.2. Peer victimization

The three items were designed for measuring peer victimization referring to Marsh et al. (2023) description of the three main domains of peer victimization including verbal, physical, and social/relational and the formulation of peer violence items in the ACE-IQ (Gette et al., 2022): “Have you been hit, kicked, or deliberately locked indoors by your peers or classmates?” “Have you been deliberately excluded from the activity by your peers or classmates, or have you been completely ignored?” and “Have you been maligned, ridiculed, or given an insulting nickname by your peers or classmates?”. Each item was scored on the 5-point Likert scale (from 1 to 5 points), which means from “never” to “always.” Higher scores indicated greater perceived exposure to peer victimization. There was Cronbach's alpha coefficient of 0.673 for the three items. The composite reliability (CR) was 0.732 using the OMEGA macro for SPSS (Hayes and Coutts, 2020), and the average variance extracted (AVE) was 0.481 (Fornell and Larcker, 1981; Tseng et al., 2006).

#### 2.2.3. Resilience

The Connor–Davidson Resilience Scale (CD-RISC) in the Chinese version was used in our study (Yu and Zhang, 2007). The 25-item Likert scale (0 “not at all,” 1 “rarely true,” 2 “sometimes true,” 3 “often true,” and 4 “extremely true”) was used to assess resilience, which includes three dimensions of tenacity (13 items, describing a person's calmness, promptness, perseverance, and sense of control in the face of difficulty and adversity, e.g., “know where to get help”), strength (8 items, concentrating on the person's potential for overcoming obstacles and growing from prior events, e.g., “able to adapt to change”), and optimism (4 items, reflecting the person's tendency to look on the positive sides of things and trusting one's personal and social resources, e.g., “close and secure relationships”) (Yu and Zhang, 2007). The higher the score, the more resilience the person has. Cronbach's alpha coefficient was 0.964 in total. The CR (AVE) of tenacity, strength, and optimism was 0.949 (0.588), 0.899 (0.530), and 0.760 (0.450), respectively.

#### 2.2.4. Quality of life

The QoL was assessed by the Adolescent Quality of Life Scale (Tang et al., 2022). The 39-item scale was divided into four dimensions to assess children's and teenagers' QoL: physical (8 items), psychological (11 items), social (14 items), and pubertal (6 items). On a 5-point Likert scale, each item received a score. When a question linked to a phenomenon's frequency was asked, it was graded on a scale of 5 to 1, which ranged from “never” to “always.” When a certain situation was asked, a satisfactory score ranged from 1 to 5 which meant “extremely unsatisfied” to “very satisfied”. Higher scores also indicated better QoL. Cronbach's alpha coefficient was 0.949 in total, and coefficients were 0.888, 0.883, 0.900, and 0.637 for physical, psychological, social, and pubertal, respectively. The CR (AVE) of physical, psychological, social, and pubertal was 0.889 (0.504), 0.878 (0.411), 0.902 (0.401), and 0.729 (0.390), respectively.

#### 2.2.5. Positive childhood experiences

The PCEs were estimated by the Benevolent Childhood Experiences Scale (BCEs) in the Chinese version (Zhan et al., 2021). Items pertained to perceived relational and internal safety and security (e.g., at least one safe caregiver and beliefs that gave comfort), positive and predictable QoL (e.g., enjoyment of school, regular meals, and bedtime), and interpersonal support (e.g., a teacher who cared and a supportive non-caregiver adult) between ages 0–18 years. The scale contains 10 items. The option “no” was scored as 0 points, and the “yes” was 1. If there are more than 8 points in the 10 items, the code is “High-PCE group”; otherwise, the code is “Low-PCE group” (Hou et al., 2022). In this study, Cronbach's alpha coefficient was 0.768. The CR was 0.767, and the AVE was 0.255.

### 2.3. Statistical analysis

Random forest imputation was performed using the package missForest 1.5 (Stekhoven and Bühlmann, 2012) in R version 4.2.1. The number of random seed was set to 123.^1^ The data were analyzed using SPSS version 26.0 and Mplus version 8.3. General demographic variables, QoL, resilience, and peer victimization were calculated in the two PCEs groups using frequency and percentage, or mean and standard deviation. The chi-squared test, *t*-test, and Mann–Whitney test were used to compare differences in the variables between the PCEs groups. The correlations between peer victimization, PCEs, resilience, and QoL were measured by Spearman's correlation. Mplus version 8.3 was used to explore the mediation effect of resilience among peer victimization and QoL or its four dimensions, as well as the moderation effect of PCEs. The differences in path coefficients between the two PCEs groups were also compared. The χ^2^*/df* below 5, comparative fit index (CFI), and Tucker–Lewis index (TLI) above 0.90 (Salisbury et al., 2002), root mean square error of approximation (RMSEA) below 0.06, and standardized root mean square (SRMR) below 0.08 indicate if the model fits well (Hu and Bentler, 1999). When at least two of the following criteria were satisfied, models fit the data equally well: Δχ^2^was not significant at a *P*-value of <0.05, ΔCFI < 0.01, or ΔRMSEA < 0.015 (Cheung and Rensvold, 2002). The path was considered significant when the two-tailed *P*-values were below 0.05 and the 95% bias-corrected bootstrap confidence intervals excluded zero. The bias-corrected bootstrap samples were 10,000. All differences were considered statistically significant at a *P*-value of <0.05.

## 3. Results

### 3.1. Common method bias test

The Harman single-factor test for the common method bias test was used (Podsakoff et al., 2003). All entries for QoL, peer victimization, resilience, and PCEs were included in the analysis. The results showed that a total of 13 components with eigenvalues >1 emerged, and the first component accounted for 31.68% of the total variance, which was much less than the critical value of 40%. Thus, the common method bias in our study was considered to be within an acceptable range.

### 3.2. Descriptive and correlation results

The details for general demographic variables grouped by PCEs are shown in Table 1. Among general demographic variables, the two PCEs groups differed in terms of school location, grade, gender, and living in school (*P* < 0.05).

**Table 1:** Differences for general demographic variables and study variables grouped by PCEs (*n* = 1,706).

**Variables**		**Low-PCEs *n* (%)/Mean (SD)**	**High-PCEs *n* (%)/Mean (SD)**	***P*-value**
School location	Qijiang	285 (16.71)	464 (27.20)	**< 0.001**
	Yubei	523 (30.66)	434 (25.44)	
Grade	7th	328 (19.23)	489 (28.66)	**< 0.001**
	10th	480 (28.14)	409 (23.97)	
Gender	Male	385 (22.57)	483 (28.31)	**< 0.05**
	Female	423 (24.79)	415 (24.33)	
Family residence	Countryside	91 (5.33)	111 (6.51)	0.072
	Township	313 (18.35)	300 (17.58)	
	City	404 (23.68)	487 (28.55)	
Single child	Yes	243 (14.24)	281 (16.47)	0.586
	No	565 (33.12)	617 (36.17)	
Living in school	Yes	433 (25.38)	367 (21.51)	**< 0.001**
	No	375 (21.98)	531 (31.13)	
QoL	Total	128.516 (20.453)	151.302 (19.784)	**< 0.001**
	Physical	26.132 (5.792)	30.719 (5.487)	**< 0.001**
	Psychological	36.348 (7.699)	42.817 (7.165)	**< 0.001**
	Social	45.259 (8.099)	54.687 (7.789)	**< 0.001**
	Pubertal	20.777 (3.160)	23.078 (2.929)	**< 0.001**
Resilience		45.892 (18.007)	65.441 (19.471)	**< 0.001**
Peer victimization		3.803 (1.698)	3.361 (1.046)	**< 0.001**

SD, standard deviation; QoL, quality of life; PCEs, positive childhood experiences. The PCEs were divided into two groups, someone who has a PCEs score over 8 points belongs to the high-PCEs group, while others are in the low-PCEs group. The chi-squared test was used for general demographic variables. The *t*-test was used for QoL and resilience, while the Mann–Whitney test was used for peer victimization because it is significantly far from the normal distribution. The bold values mean that the *P*-values were significant.

The scores of QoL and its four dimensions, resilience, and peer victimization were also compared between the two PCEs groups as shown in Table 1. In our sample, the mean total QoL score was 128.516 (standard deviation, SD = 20.453) in the low-PCEs group vs. 151.302 (SD = 19.784) in the high-PCEs group. The mean and standard deviation of physical, psychological, social, and pubertal QoL were 26.132 (SD = 5.792), 36.348 (SD = 7.699), 45.259 (SD = 8.099), and 20.777 (SD = 3.160) in the low-PCEs group and were 30.719 (SD = 5.487), 42.817 (SD = 7.165), 54.687 (SD = 7.789), and 23.078 (SD = 2.929) in the high-PCEs group. The mean scores of total QoL and the four dimensions were significantly different between the two PCEs groups (*P* < 0.001). Resilience and peer victimization were also found to be different in the two PCEs groups (*P* < 0.001). The mean score of resilience was 45.892 (SD = 18.007) in the low-PCEs group vs. 65.441 (SD = 19.471) in the high-PCEs group. The mean score of peer victimization was 3.803 (SD = 1.698) in the former vs. 3.361 (SD = 1.046) in the latter.

Spearman's correlations were used because the study variables did not conform to a normal distribution. Detailed correlation coefficients in the two PCEs groups are shown in Table 2, with the coefficients for the low-PCEs group and the high-PCEs group located in the lower and upper triangular parts, respectively. Peer victimization was negatively correlated with QoL, four dimensions of QoL, and resilience. Resilience was positively correlated with the total and four dimensions of QoL. All coefficients were significant (*P* < 0.001). It is worth mentioning that peer victimization and resilience in the two groups had correlation coefficients below 0.5, and there was little multicollinearity between the two groups (Grewal et al., 2004).

**Table 2:** Spearman's correlation coefficients grouped by the two PCEs groups.

**High-PCEs**	**1**	**2**	**3**	**4**	**5**	**6**	**7**	**8**
**Low-PCEs**								
1 Total QoL		0.818^**^	0.875^**^	0.885^**^	0.694^**^	0.614^**^	−0.300^**^	0.254^**^
2 Physical	0.777^**^		0.698^**^	0.589^**^	0.413^**^	0.419^**^	−0.230^**^	0.211^**^
3 Psychological	0.861^**^	0.618^**^		0.645^**^	0.466^**^	0.493^**^	−0.259^**^	0.208^**^
4 Social	0.874^**^	0.547^**^	0.643^**^		0.677^**^	0.613^**^	−0.298^**^	0.250^**^
5 Pubertal	0.558^**^	0.328^**^	0.330^**^	0.482^**^		0.553^**^	−0.174^**^	0.188^**^
6 Total Resilience	0.423^**^	0.229^**^	0.360^**^	0.434^**^	0.320^**^		−0.210^**^	0.267^**^
7 Peer victimization	−0.368^**^	−0.235^**^	−0.336^**^	−0.363^**^	−0.129^**^	−0.117^**^		−0.093^**^
8 PCEs scores	0.393^**^	0.246^**^	0.278^**^	0.433^**^	0.284^**^	0.384^**^	−0.120^**^	

** *P* < 0.001. Physical, psychological, social, and pubertal were dimensions of QoL. The coefficients of the low-PCEs group and the high-PCEs group were located in the lower and upper triangular parts, respectively.

### 3.3. Multiple-group mediation analysis

Grade, gender, and family residence were found to be associated with QoL and its dimensions, resilience, and peer victimization in this study. So the three general demographic variables were included as covariates in the mediation models for control. All unconstrained mediation models were saturated (χ^2^ = 0.000, *df* = 0, *P* = 0.000, CFI = 1.000, RMSEA = 0.000, SRMR = 0.000).

**Figure 1:**
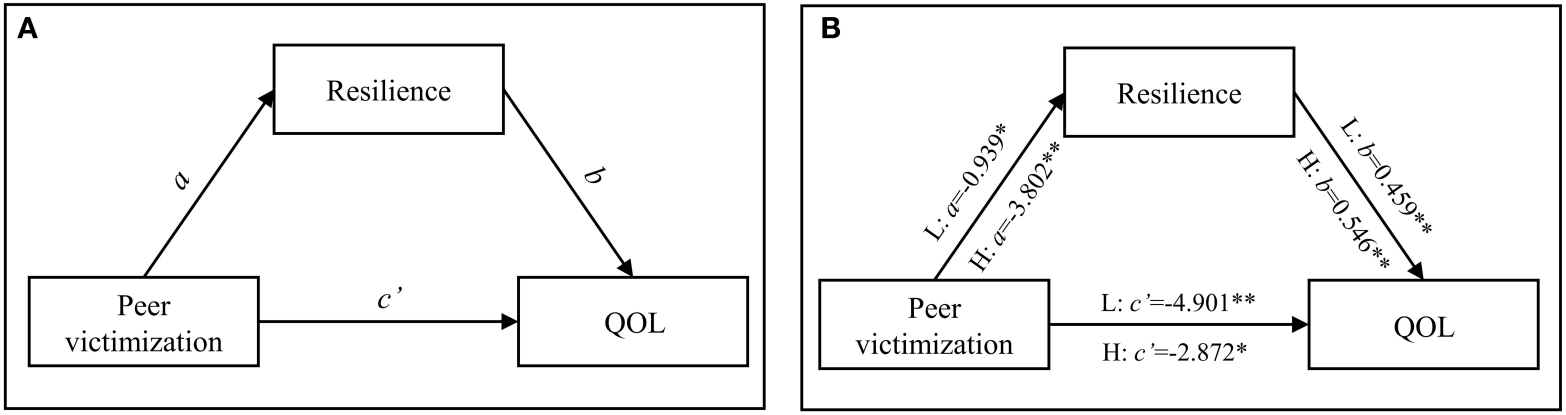
Mediation of resilience between peer victimization and QoL in the two PCEs groups. **(A)** Was the hypothesized model of peer victimization on resilience and QOL. **(B)** Showed the coefficients of the total QOL mediation mechanism. L, low-PCEs group; H, high-PCEs group. The coefficients were shown in unstandardized regression coefficients. ^**^P < 0.001, ^*^P < 0.05.

**Figure 2:**
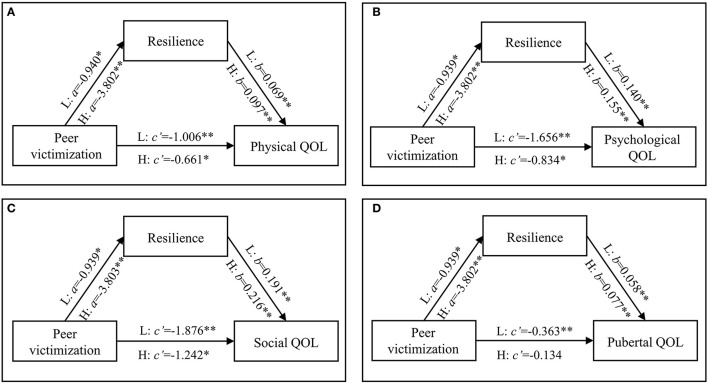
Mediation models with four dimensions of QOL as dependent variables in the two PCEs groups. **(A–D)** Were physical, psychological, social, and pubertal QOL as the dependent variables, respectively. L, low-PCEs group; H, high-PCEs group. The coefficients were shown in unstandardized regression coefficients. ^**^P < 0.001, ^*^P < 0.05.

For the mediation model with QoL as the dependent variable in the two PCEs groups, the model comparison test showed a significant deterioration in the model with constrained path coefficients compared to the unconstrained model (Δχ^2^*/df* was significant at *P* < 0.001, ΔCFI = 0.027, ΔRMSEA = 0.082). Similarly, in models with the four dimensions of QoL as the dependent variables, the constrained models also showed poorer fit results (Δχ^2^*/df* was significant at *P* < 0.05, ΔCFI > 0.01, ΔRMSEA > 0.015). It might show that the PCEs did affect the relationship among peer victimization, resilience, and QoL. Therefore, we ultimately chose the unconstrained models for analysis. The mediation mechanisms under the two-level PCEs are shown in Figures 1, 2. The detailed 95% confidence interval and the significance of the mediating path coefficients are indicated in Table 3.

**Table 3:** Unstandardized model coefficients of mediation in the two PCEs groups after controlling for covariates.

**Dependents**	**Predictors**	**Low–PCEs**			**High–PCEs**		
		**Estimate**	***P***	**BC95%*CI***	**Estimate**	***P***	**BC95%*CI***
QoL	Total effect	−5.332	**< 0.001**	(−6.186, −4.557)	−4.949	**< 0.001**	(−6.982, −2.687)
	Indirect	−0.431	**< 0.05**	(−0.833, −0.086)	−2.077	**< 0.001**	(−2.863, −1.443)
	Direct	−4.901	**< 0.001**	(−5.634, −4.243)	−2.872	**< 0.01**	(−4.734, −0.889)
Physical QOL	Total effect	−1.071	**< 0.001**	(−1.351, −0.795)	−1.031	**< 0.001**	(−1.530, −0.512)
	Indirect	−0.065	**< 0.05**	(−0.136, −0.014)	−0.370	**< 0.001**	(−0.526, −0.247)
	Direct	−1.006	**< 0.001**	(−1.272, −0.738)	−0.661	**< 0.05**	(−1.147, −0.174)
Psychological QOL	Total effect	−1.788	**< 0.001**	(−2.087, −1.498)	−1.423	**< 0.001**	(−2.107, −0.703)
	Indirect	−0.132	**< 0.05**	(−0.259, −0.027)	−0.589	**< 0.001**	(−0.824, −0.400)
	Direct	−1.656	**< 0.001**	(−1.939, −1.376)	−0.834	**< 0.05**	(−1.475, −0.186)
Social QOL	Total effect	−2.056	**< 0.001**	(−2.414, −1.690)	−2.065	**< 0.001**	(−2.864, −1.190)
	Indirect	−0.180	**< 0.05**	(−0.347, −0.036)	−0.823	**< 0.001**	(−1.134, −0.571)
	Direct	−1.876	**< 0.001**	(−2.190, −1.570)	−1.242	**< 0.01**	(−1.960, −0.462)
Pubertal QOL	Total effect	−0.417	**< 0.001**	(−0.595, −0.230)	−0.429	**< 0.01**	(−0.678, −0.190)
	Indirect	−0.054	**< 0.05**	(−0.109, −0.011)	−0.295	**< 0.001**	(−0.404, −0.208)
	Direct	−0.363	**< 0.001**	(−0.530, −0.190)	−0.134	0.241	(−0.365, 0.079)

Total effect: Peer victimization → QoL + Peer victimization → Resilience → QoL. Indirect: Peer victimization → Resilience → QoL. Direct: Peer victimization → QoL. BC 95% *CI* is bias-corrected bootstrap 95% confidence interval. The bold values mean that the *P*-values were significant.

Figure 1 and Table 3 show the hypothesized model and path coefficients when QoL was the dependent variable. In the two PCEs groups, peer victimization negatively affected QoL not only through a direct effect but also an indirect effect *via* resilience. In the low-PCEs group, the direct effect of peer victimization on QoL was −4.901 (*P* < 0.001, 91.92% of the total effect), and the indirect effect was −0.431 (*P* < 0.05, 8.08% of the total effect). In the high-PCEs group, the direct effect of peer victimization was −2.872 (*P* < 0.01, 58.03% of the total effect), and the indirect effect through resilience was −2.077 (*P* < 0.001, 41.97% of the total effect).

Figure 2 and Table 3 show the mediation models with the four dimensions of QoL as the dependent variables. The path coefficients of peer victimization to resilience were from −0.939 to −0.940 in the low-PCEs group and from −3.802 to −3.803 in the high-PCEs group. As shown in Figure 2, the direct effects of peer victimization on four dimensions of QoL in the low-PCEs group took values ranging from −0.363 to −1.876, all of which were statistically significant (*P* < 0.05). The direct path coefficients in the high-PCEs group ranged from −0.134 to −1.242, where all direct path coefficients were significant (*P* < 0.05) except the path coefficient of pubertal QoL. The indirect effect values for the low-PCEs group ranged from −0.054 to −0.180 (*P* < 0.05), accounting for 6.07–12.95% of the total effect, while the high-PCEs group had indirect effect values ranging from −0.295 to −0.823 (*P* < 0.001), accounting for 35.89–68.76% of the total effect.

Finally, the results of the differences in path coefficients between the two PCEs groups are shown in Table 4. The total effect coefficients (path *c*) were not significantly different between the two PCEs groups in all models (*P* > 0.05). Except for psychological QoL, the direct effect coefficients (path *c'*) of the mediation models were not significantly different between the two PCEs groups (*P* > 0.05). The differences in path *a* (peer victimization to resilience) between the two groups were significant in all models (*P* < 0.05). The difference in path *b* (resilience to QoL) was significant only in the model with pubertal QoL as the dependent variable (*P* < 0.05). Differences in indirect effects (path a^*^b) were significant in all models (*P* < 0.05).

**Table 4:** Differences in path coefficients compared between the two PCEs groups.

**Dependent Variable**	**Difference** ***c***		**Difference** ***c'***		**Difference a**		**Difference** ***b***		**Difference** a^*^b	
	**Estimate**	***P***	**Estimate**	***P***	**Estimate**	***P***	**Estimate**	***P***	**Estimate**	***P***
QOL	−0.384	0.749	−2.029	0.059	2.863	**< 0.001**	−0.087	0.089	1.646	**< 0.001**
Physical QOL	−0.040	0.895	−0.345	0.234	2.862	**< 0.001**	−0.028	0.071	0.305	**< 0.001**
Psychological QOL	−0.365	0.362	−0.822	**< 0.05**	2.863	**< 0.001**	−0.014	0.478	0.457	**< 0.001**
Social QOL	0.009	0.984	−0.634	0.133	2.864	**< 0.001**	−0.025	0.220	0.643	**< 0.001**
Pubertal QOL	0.012	0.940	−0.229	0.111	2.863	**< 0.001**	−0.020	**< 0.05**	0.241	**< 0.001**

Difference *c*, path *c* in low-PCEs group minus *c* in high-PCEs group; difference *c'*, path *c'* in low-PCEs group minus *c'* in the high-PCEs group; difference *a*, path *a* in the low-PCEs group minus *a* in the high-PCEs group; difference *b*, path *b* in the low-PCEs group minus *b* in the high-PCEs group; difference a^*^b, indirect effect in the low-PCEs group minus which in the high-PCEs group. The bold values mean that the *P*-values were significant.

## 4. Discussion

Our study was the first to examine the effects of peer victimization on adolescents' QoL and the potential mediating mechanisms from the perspective of PCEs. The results showed that peer victimization had a negative correlation with adolescents' QoL, four dimensions of QoL, and resilience, while resilience had a positive correlation with QoL and four dimensions of QoL. These correlations remained significant across different PCEs groups. It was shown that resilience could partially mediate the link between peer victimization and QoL at two levels of PCEs, with similar direct effects and different indirect effects. Overall, these findings were consistent with our hypothesis that traumatic experiences such as peer victimization negatively affect QoL. Resilience partially mediated this relationship. PCEs moderated the relationship among the three.

Resilience was negatively associated with peer victimization and positively associated with QoL and its four dimensions, which was similar to the results of previous studies (Ran et al., 2020; Anderson et al., 2022; Martín-Pérez et al., 2022; Tang et al., 2022). Therefore, it was reasonable to assume that peer victimization may predict poorer QoL *via* decreasing resilience. Similar to previous studies (Anderson et al., 2022; Tang et al., 2022), the results of this study showed that resilience partially mediated the effect of peer victimization on QoL and its three dimensions except for pubertal QoL as reflected in the mediation models. On the one hand, the indirect effect of resilience on QoL was significant among middle school students. It meant resilience could help individuals buffer some of the negative effects on QoL when peer victimization seemed unavoidable. Resilience, as a result of an individual's continuous adaptation to the environment, helped individuals recover from adversity and promote positive development (Chen et al., 2021). There were a wealth of interventions on resilience, such as the empower resilience intervention (ERI) used by Chandler et al. (2015), which intervened with subjects in the areas of emotion, cognition, physical health, social support, and positive thinking and was able to reduce health risk behaviors and physical and psychological symptoms. A recent review showed that interventions targeting adolescents/children/adults (especially those exposed to trauma) and interventions in the form of positive thinking, psychoeducation, and social support can have significant positive effects (Liu et al., 2020). These suggested the feasibility of intervention from resilience for buffering peer victimization in future. On the other hand, this partial mediating effect also meant that peer victimization can affect QoL via other factors. When the possibility of peer victimization cannot be completely eliminated, it is necessary to further explore other mediating roles in future.

Differences in scores of peer victimization, resilience, and QoL were found between the two groups of PCEs. The presence of PCEs can also influence the association among the three variables, as reflected in the mediation models. The findings of this study demonstrated an association between increased peer victimization and poorer perceived QoL across the two PCEs groups, which were consistent with previous studies (Sumter et al., 2012; Chen et al., 2018; Forbes et al., 2019). However, there were no significant differences in the total effects of peer victimization on QoL or its four dimensions between the two PCEs groups. This result differed from some literature on the role of PCEs (Yin et al., 2017; Hou et al., 2022), which suggested that PCEs attenuate the effects of peer victimization. But it is similar to results of Averdijk et al. (2014), which found that the interaction between peer victimization and social support did not have a significant protection for internalizing problems. It could be found that the direct effects in the high-PCEs group were smaller than those in the low-PCEs group, but only the difference in the models of the psychological dimension of QoL was significant. This might suggest that increased PCEs have a more pronounced buffering effect on negative psychological consequences caused by peer victimization. The indirect effects of peer victimization on QoL and its four dimensions were significantly larger in the high-PCEs group than in the low-PCEs group. This result might indicate that PCEs mainly moderated the indirect path. It means that increasing the number of PCEs in adolescents could enhance the effectiveness of resilience interventions for peer victimization. It was possible to explain that the impact on QoL was manageable even with the risk of peer victimization because people in the high-PCEs group might have had more resources (Cohen and Wills, 1985) or more appropriate coping methods (Zheng et al., 2022) to combat the risks associated with peer victimization.

As for specific path coefficients, the effects of peer victimization on resilience (path *a*) have increased in the high-PCEs group, which meant the PCEs mainly affect this pathway. The specificity of the association between peer victimization and resilience had similar results in previous literature. Several studies showed that there were significant negative associations between ACEs and numerous physical, psychological, and social health outcomes and life satisfaction at higher PCEs, whereas these relationships were not significant at lower PCEs (Crandall et al., 2019; Xu et al., 2022). Some speculations were as follows: people with fewer PCEs already had adverse mental health conditions and would not be further affected by ACEs, but those with more PCEs faced more dramatic health shifts when ACEs occurred due to better baseline health conditions or felt more shame and were reluctant to talk about (Crandall et al., 2019). The effects of resilience to QoL and its four dimensions (path *b*) in the high-PCEs group were slightly larger than those in the low-PCEs group. Although the difference was not significant except for the model of pubertal QoL. Higher PCEs could be used as a promoting variable to develop resilience (Zimmerman et al., 2013; Narayan et al., 2018) and then contribute to enhancing an individual's health, which might explain the relationship between resilience and QoL in higher PCEs. Though the role of PCEs was relatively complex, its total effect was to help promote better QoL. Therefore, in future, more attention should be paid to PCEs when intervening against peer victimization. Some studies have explored interventions to increase PCEs, such as the 2017 HOPE (Health Outcomes From Positive Experiences) project, which aimed to prevent ACEs and other risky environmental variables and increase four types of PCEs: supportive relationships, safe and equitable environments, social engagement, and social and emotional competence (Sege and Harper Browne, 2017).

### 4.1. Study limitations

In interpreting the findings of our study, several limitations need to be taken into consideration. First, the sample involved only limited representativeness of adolescents because the sample was restricted to students in the 7th and 10th grades of two districts in Chongqing, China. Second, the data were cross-sectional, where causal associations between the measured variables cannot be established. Third, the collected data from the cohort study were based on self-reports, which were based on subjective criteria and were vulnerable to intentional or even unconscious reporting errors. Peer victimization was measured by self-designed items rather than scientific scales, which can only capture an approximate level of peer victimization and would result in partial loss of information. The low reliability of the pubertal dimension of the Adolescent Quality of Life Scale may have caused some bias in the results. Finally, there were numerous factors influencing QoL, but the limited covariates controlled in this study may have caused some bias.

### 4.2. Conclusion

In our study, peer victimization had a significant negative correlation with adolescents' QoL and resilience, while resilience had a positive correlation with QoL. These correlations remained significant across different PCEs groups. Resilience was found to partially mediate the effect between peer victimization and quality of life across the two PCEs groups in Chongqing adolescents. The indirect effect of resilience was stronger in the group with higher positive childhood experiences, while the direct effect was slightly weaker in the high-PCEs group than in the low-PCEs group. When peer victimization is unavoidable, schools, families, and society should focus on resilience intervention and prioritize the enhancement of positive childhood experiences in terms of improving the quality of life for youth.

For example, schools could choose strategies, such as training current teachers' psychological skills, hiring professional psychological teachers, increasing the number of mental health courses, or enhancing psychological counseling, according to their financial situation (rural and urban schools might be different in it). At the same time, schools need to supervise whether there is any bullying behavior on campus through students and teachers and impose appropriate punishments on the perpetrators of bullying. Parents could actively seek knowledge about adolescent mental health from schools and the media, not engage in risky behaviors such as gambling or excessive drinking, and foster open and comprehensive communications within the family. For society, it may be a good choice to report the negative effects and prevention methods of peer bullying. During the promotion process, attention should be paid to protecting the reputation and privacy of teenagers to prevent secondary harm.

## Data availability statement

The raw data supporting the conclusions of this article will be made available by the corresponding author, without undue reservation.

## Ethics statement

The studies involving human participants were reviewed and approved by the Ethics Committee of Chongqing Medical University. Written informed consent to participate in this study was provided by the participants' legal guardian/next of kin.

## Author contributions

LD participated in work design, research investigation, data collection and analysis, and original manuscript drafting and revising. YL participated in work design, data analysis, and critically revising important intellectual content. HW contributed to work design, critically revising important intellectual content, and funding acquisition. JY and LL participated in work design, research investigation, and data collection. All authors read and approved the final manuscript.

## Footnotes

^1^Random forest imputation is a statistical method for filling missing data. It uses random forest algorithm to predict missing values by using information from other features and fill in missing items in the dataset. The random number seeds can be arbitrarily selected (123 and 1234 are both okay). The purpose of setting a random number seed as 123 is to ensure that the imputation results can be replicated.

## References

[B1] Almuneef M. ElChoueiry N. Saleheen H. Al-Eissa M. (2018). The impact of Adverse Childhood Experiences on social determinants among Saudi adults. J. Public Health 40, e219–e227. 10.1093/pubmed/fdx177 29294073

[B2] Anderson J. R. Mayes T. L. Fuller A. Hughes J. L. Minhajuddin A. Trivedi M. H. (2022). Experiencing bullying's impact on adolescent depression and anxiety: Mediating role of adolescent resilience. J. Affect. Disord. 310, 477–483. 10.1016/j.jad.2022.04.003 35390356

[B3] Averdijk M. Eisner M. Ribeaud D. (2014). Do social relationships protect victimized children against internalizing problems? J. Sch. Violence 13, 80–99. 10.1080/15388220.2013.84217529473091

[B4] Blakemore S. J. Mills K. L. (2014). Is adolescence a sensitive period for sociocultural processing? Annu. Rev. Psychol. 65, 187–207. 10.1146/annurev-psych-010213-115202 24016274

[B5] Blosnich J. Bossarte R. (2011). Low-level violence in schools: is there an association between school safety measures and peer victimization? J. Sch Health 81, 107–113. 10.1111/j.1746-1561.2010.00567.x 21223278

[B6] Chan H. C. Wong D. S. W. (2015). Traditional school bullying and cyberbullying in Chinese societies: Prevalence and a review of the whole-school intervention approach. Aggress. Violent Behav. 23, 98–108. 10.1016/j.avb.2015.05.010

[B7] Chandler G. E. Roberts S. J. Chiodo L. (2015). Resilience intervention for young adults with adverse childhood experiences. J. Am. Psych. Nurses Assoc. 21, 406–416. 10.1177/1078390315620609 26711904

[B8] Chen Q. Chen M. Zhu Y. Chan K. L. Ip P. (2018). Health correlates, addictive behaviors, and peer victimization among adolescents in China. World J. Pediatr. 14, 454–460. 10.1007/s12519-018-0158-2 29956126

[B9] Chen Y. Hua K. Huang C. Zhou G. Wang J. (2021). Adverse childhood experiences and psychological well-being in chinese college students: moderated mediation by gender and resilience. Front. Psychiatry 12, 710635. 10.3389/fpsyt.2021.710635 34434130 PMC8381021

[B10] Cheung G. W. Rensvold R. B. (2002). Evaluating goodness-of-fit indexes for testing measurement invariance. Struct. Equ. Model. 9, 233–255. 10.1207/S15328007SEM0902_5

[B11] Cohen S. Wills T. A. (1985). Stress, social support, and the buffering hypothesis. Psychol. Bull. 98, 310–357. 10.1037/0033-2909.98.2.310 3901065

[B12] Crandall A. Miller J. R. Cheung A. Novilla L. K. Glade R. Novilla M. L. B.. (2019). ACEs and counter-ACEs: How positive and negative childhood experiences influence adult health. Child Abuse Negl. 96, 104089. 10.1016/j.chiabu.2019.104089 31362100

[B13] Cruz D. Collet N. Nóbrega V. M. (2018). Quality of life related to health of adolescents with dm1: an integrative review. Cienc. Saude Coletiva 23, 973–989. 10.1590/1413-81232018233.08002016 29538577

[B14] Díaz K. I. Fite P. J. Abel M. R. Doyle R. L. (2021). Varying experiences of cyber victimization among middle and high school students. Child Youth Care Forum 50, 1087–1105. 10.1007/s10566-021-09614-4 33879985 PMC8051549

[B15] Forbes M. K. Fitzpatrick S. Magson N. R. Rapee R. M. (2019). Depression, anxiety, and peer victimization: bidirectional relationships and associated outcomes transitioning from childhood to adolescence. J. Youth Adolesc. 48, 692–702. 10.1007/s10964-018-0922-6 30229362 PMC6447080

[B16] Fornell C. Larcker D. (1981). Evaluating structural equation models with unobservable variables and measurement error. J. Mark. Res. 24, 337–346. 10.1177/002224378101800104

[B17] Gette J. A. Gissandaner T. D. Littlefield A. K. Simmons C. S. Schmidt A. T. (2022). Modeling the adverse childhood experiences questionnaire-international version. Child Maltreat. 27, 527–538. 10.1177/10775595211043122 34569305

[B18] Gower T. Rancher C. Campbell J. Mahoney A. Jackson M. McDonald R.. (2020). Caregiver and divine support: Associations with resilience among adolescents following disclosure of sexual abuse. Child Abuse Negl. 109, 104681. 10.1016/j.chiabu.2020.104681 32919169

[B19] Grewal R. Cote J. Baumgartner H. (2004). Multicollinearity and measurement error in structural equation models: Implications for theory testing. Mark. Sci. 23, 519–529. 10.1287/mksc.1040.0070

[B20] Han Z. Zhang G. Zhang H. (2017). School bullying in urban china: prevalence and correlation with school climate. Int. J. Environ. Res. Public Health 14, 1116. 10.3390/ijerph14101116 28946682 PMC5664617

[B21] Hayes A. F. Coutts J. J. (2020). Use omega rather than cronbach's alpha for estimating reliability. But... Commun. Methods Meas. 14, 1–24. 10.1080/19312458.2020.1718629

[B22] Helseth S. Misvaer N. (2010). Adolescents' perceptions of quality of life: what it is and what matters. J. Clin. Nurs. 19, 1454–1461. 10.1111/j.1365-2702.2009.03069.x 20500355

[B23] Herman J. L. (1992). Trauma and Recovery. New York: Basic Books.

[B24] Herzer M. Zeller M. H. Rausch J. R. Modi A. C. (2011). Perceived social support and its association with obesity-specific health-related quality of life. J. Dev. Behav. Pediatr. 32, 188–195. 10.1097/DBP.0b013e318208f576 21263350 PMC3480181

[B25] Hofhuis J. G. M. van Stel H. F. Schrijvers A. J. P. Rommes J. H. Bakker J. Spronk P. E. (2009). Health-related quality of life in critically ill patients: how to score and what is the clinical impact? Curr. Opin. Crit. Care 15, 425–430. 10.1097/MCC.0b013e32833079e4 19623059

[B26] Hogg F. R. A. Peach G. Price P. Thompson M. M. Hinchliffe R. J. (2012). Measures of health-related quality of life in diabetes-related foot disease: a systematic review. Diabetologia 55, 552–565. 10.1007/s00125-011-2372-5 22246373

[B27] Hou H. Zhang C. Tang J. Wang J. Xu J. Zhou Q.. (2022). Childhood experiences and psychological distress: can benevolent childhood experiences counteract the negative effects of adverse childhood experiences? Front. Psychol. 13, 800871. 10.3389/fpsyg.2022.800871 35282200 PMC8914177

[B28] Hu L. Bentler P. M. (1999). Cutoff criteria for fit indexes in covariance structure analysis: Conventional criteria versus new alternatives. Struct. Equ. Modeling 6, 1–55. 10.1080/1070551990954011836787513

[B29] Jantzer V. Ossa F. C. Eppelmann L. Parzer P. Resch F. Kaess M. (2022). Under the skin: does psychiatric outcome of bullying victimization in school persist over time? A prospective intervention study. J. Child Psychol. Psychiatry 63, 646–654. 10.1111/jcpp.13502 34396522

[B30] Kaczmarek C. Haller D. M. Yaron M. (2016). Health-related quality of life in adolescents and young adults with polycystic ovary syndrome: a systematic review. J. Pediatr Adolesc. Gynecol. 29, 551–557. 10.1016/j.jpag.2016.05.006 27262833

[B31] Lee S. Lee J. Choi J. Y. (2017). The effect of a resilience improvement program for adolescents with complex congenital heart disease. Eur. J. Cardiovasc. Nurs. 16, 290–298. 10.1177/1474515116659836 27400701

[B32] Lerner J. V. Phelps E. Forman Y. E. Bowers E. P. (2009). “Positive youth development,” in Handbook of Adolescent Psychology: Individual bases of Adolescent Development. (Hoboken, NJ: John Wiley and Sons, Inc.) 524–558. 10.1002/9780470479193.adlpsy001016

[B33] Liu J. J. W. Ein N. Gervasio J. Battaion M. Reed M. Vickers K. (2020). Comprehensive meta-analysis of resilience interventions. Clin. Psychol. Rev. 82, 101919. 10.1016/j.cpr.2020.101919 33045528

[B34] Lo C. K.-M. Ho F. K.-W. Yan E. Lu Y. Chan K. L. Ip P. (2021). Associations between child maltreatment and adolescents' health-related quality of life and emotional and social problems in low-income families, and the moderating role of social support. J. Interpers. Violence 36, 7436–7455. 10.1177/0886260519835880 30862240

[B35] Maheri M. Alipour M. Rohban A. Garmaroudi G. (2019). The association of resilience with health-related quality of life (HRQoL) in adolescent students. Int. J. Adolesc. Med. Health 34, 20190050. 10.1515/ijamh-2019-0050 31665118

[B36] Malibary H. Zagzoog M. M. Banjari M. A. Bamashmous R. O. Omer A. R. (2019). Quality of Life (QoL) among medical students in Saudi Arabia: a study using the WHOQOL-BREF instrument. BMC Med. Educ. 19, 344. 10.1186/s12909-019-1775-8 31500610 PMC6734217

[B37] Marçal K. E. Maguire-Jack K. (2022). Informal supports, housing insecurity, and adolescent outcomes: Implications for promoting resilience. Am. J. Community Psychol. 70, 178–196. 10.1002/ajcp.12589 35156209

[B38] Marengo D. Settanni M. Prino L. E. Parada R. H. Longobardi C. (2019). Exploring the dimensional structure of bullying victimization among primary and lower-secondary school students: is one factor enough, or do we need more? Front. Psychol. 10, 770. 10.3389/fpsyg.2019.00770 31019481 PMC6458248

[B39] Marsh H. W. Reeve J. Guo J. Pekrun R. Parada R. H. Parker P. D.. (2023). Overcoming limitations in peer-victimization research that impede successful intervention: challenges and new directions. Perspect. Psychol. Sci. 18, 812–828. 10.1177/17456916221112919 36239467 PMC10336717

[B40] Martín-Pérez Á. D. L. Morán-Sánchez I. Gascón-Cánovas J.J. (2022). The impact of resilience as a protective factor on Health-Related Quality of Life's psychological dimensions among adolescents who experience peer victimization. Sci. Rep 12, 18898. 10.1038/s41598-022-23424-1 36344809 PMC9640611

[B41] Menesini E. Salmivalli C. (2017). Bullying in schools: the state of knowledge and effective interventions. Psychol. Health Med. 22, 240–253. 10.1080/13548506.2017.1279740 28114811

[B42] Mikkelsen H. T. Småstuen M. C. Haraldstad K. Helseth S. Skarstein S. Rohde G. (2022). Changes in health-related quality of life in adolescents and the impact of gender and selected variables: a two-year longitudinal study. Health Qual. Life Outcomes 20, 123. 10.1186/s12955-022-02035-4 35982467 PMC9387404

[B43] Modecki K. L. Minchin J. Harbaugh A. G. Guerra N. G. Runions K. C. (2014). Bullying prevalence across contexts: a meta-analysis measuring cyber and traditional bullying. J. Adolesc Health 55, 602–611. 10.1016/j.jadohealth.2014.06.007 25168105

[B44] Narayan A. J. Rivera L. M. Bernstein R. E. Harris W. W. Lieberman A. F. (2018). Positive childhood experiences predict less psychopathology and stress in pregnant women with childhood adversity: A pilot study of the benevolent childhood experiences (BCEs) scale. Child Abuse Negl. 78, 19–30. 10.1016/j.chiabu.2017.09.022 28992958

[B45] Natvig G. K. Albrektsen G. Qvarnstrøm U. (2001). School-related stress experience as a risk factor for bullying behavior. J. Youth Adolesc. 30, 561–575. 10.1023/A:101044860483828006715

[B46] Nobari H. Fashi M. Eskandari A. Villafaina S. Murillo-Garcia A. Perez-Gomez J. (2021). Effect of COVID-19 on health-related quality of life in adolescents and children: a systematic review. Int. J. Environ. Res. Public Health 18, 12. 10.3390/ijerph18094563 33923120 PMC8123423

[B47] Patton G. C. Sawyer S. M. Santelli J. S. Ross D. A. Afifi R. Allen N. B.. (2016). Our future: a Lancet commission on adolescent health and wellbeing. Lancet 387, 2423–2478 10.1016/S0140-6736(16)00579-1 27174304 PMC5832967

[B48] Podsakoff P. M. MacKenzie S. B. Lee J.-Y. Podsakoff N. P. (2003). Common method biases in behavioral research: A critical review of the literature and recommended remedies. J. Appl. Psychol. 88, 879–903. 10.1037/0021-9010.88.5.879 14516251

[B49] Ran H. Cai L. He X. Jiang L. Wang T. Yang R.. (2020). Resilience mediates the association between school bullying victimization and self-harm in Chinese adolescents. J. Affect. Disord. 277, 115–120. 10.1016/j.jad.2020.07.136 32810666

[B50] Reijntjes A. Kamphuis J. H. Prinzie P. Telch M. J. (2010). Peer victimization and internalizing problems in children: a meta-analysis of longitudinal studies. Child Abuse Negl. 34, 244–252. 10.1016/j.chiabu.2009.07.009 20304490

[B51] Salisbury W. D. Chin W. W. Gopal A. Newsted P. R. (2002). Research report: Better theory through measurement - Developing a scale to capture consensus on appropriation. Inf. Syst. Res. 13, 91–103. 10.1287/isre.13.1.91.93

[B52] Sege R. D. Harper Browne C. (2017). Responding to ACEs With HOPE: health outcomes from positive experiences. Acad. Pediatr. 17, S79–S85. 10.1016/j.acap.2017.03.007 28865664

[B53] Singstad M. T. Wallander J. L. Greger H. K. Lydersen S. Kayed N. S. (2021). Perceived social support and quality of life among adolescents in residential youth care: a cross-sectional study. Health Qual. Life Outcomes 19, 29. 10.1186/s12955-021-01676-1 33482810 PMC7821657

[B54] Steineck A. Bradford M. C. Lau N. Scott S. Yi-Frazier J. P. Rosenberg A. R. (2019). A psychosocial intervention's impact on quality of life in AYAS with cancer: a post hoc analysis from the promoting resilience in stress management (PRISM) randomized controlled trial. Children-Basel 6, 124. 10.3390/children6110124 31684055 PMC6915541

[B55] Stekhoven D. J. Bühlmann P. (2012). MissForest–non-parametric missing value imputation for mixed-type data. Bioinformatics 28, 112–118. 10.1093/bioinformatics/btr597 22039212

[B56] Sumter S. R. Baumgartner S. E. Valkenburg P. M. Peter J. (2012). Developmental trajectories of peer victimization: off-line and online experiences during adolescence. J. Adolesc Health 50, 607–613. 10.1016/j.jadohealth.2011.10.251 22626488

[B57] Tang Y. Ma Y. Zhang J. Wang H. (2022). The relationship between negative life events and quality of life in adolescents: Mediated by resilience and social support. Front. Public Health 10, 980104. 10.3389/fpubh.2022.980104 36211680 PMC9538389

[B58] Tankersley A. P. Grafsky E. L. Dike J. Jones R. T. (2021). Risk and resilience factors for mental health among transgender and gender nonconforming (TGNC) youth: a systematic review. Clin. Child Fam. Psychol. Rev. 24, 183–206. 10.1007/s10567-021-00344-6 33594611

[B59] Trask P. C. Hsu M. A. McQuellon R. (2009). Other paradigms: health-related quality of life as a measure in cancer treatment its importance and relevance. Cancer J. 15, 435–440. 10.1097/PPO.0b013e3181b9c5b9 19826365

[B60] Tseng W. T. Dornyei Z. Schmitt N. (2006). A new approach to assessing strategic learning: The case of self-regulation in vocabulary acquisition. Appl. Lingusit 27, 78–102. 10.1093/applin/ami046

[B61] Turanovic J. J. (2022). Exposure to violence and victimization: reflections on 25 years of research from the national longitudinal study of adolescent to adult health. J. Adolesc. Health 71, S14–S23. 10.1016/j.jadohealth.2022.08.015 36404015

[B62] Víllora B. Larrañaga E. Yubero S. Alfaro A. Navarro R. (2020). Relations among poly-bullying victimization, subjective well-being and resilience in a sample of late adolescents. Int. J. Environ. Res. Public Health 17, 590. 10.3390/ijerph17020590 31963323 PMC7013502

[B63] Wu X. Y. Yin W. Q. Sun H. W. Yang S. X. Li X. Y. Liu H. Q. (2019). The association between disordered eating and health-related quality of life among children and adolescents: A systematic review of population-based studies. PLoS ONE 14, 17. 10.1371/journal.pone.0222777 31584956 PMC6777752

[B64] Wu Y. Zhang Y. Y. Zhang Y. T. Zhang H. J. Long T. X. Zhang Q.. (2023). Effectiveness of resilience-promoting interventions in adolescents with diabetes mellitus: a systematic review and meta-analysis. World J. Pediatr. 19, 323–339. 10.1007/s12519-022-00666-7 36534296 PMC9761642

[B65] Xu Z. Zhang D. Ding H. Zheng X. Lee R. C.-M. Yang Z.. (2022). Association of positive and adverse childhood experiences with risky behaviours and mental health indicators among Chinese university students in Hong Kong: an exploratory study. Eur. J. Psychotraumatol. 13, 2065429. 10.1080/20008198.2022.2065429 35646294 PMC9135422

[B66] Yin X.-Q. Wang L.-H. Zhang G.-D. Liang X.-B. Li J. Zimmerman M. A.. (2017). The promotive effects of peer support and active coping on the relationship between bullying victimization and depression among chinese boarding students. Psychiat. Res. 256, 59–65. 10.1016/j.psychres.2017.06.037 28623769

[B67] Yu X. Zhang J. (2007). Factor analysis and psychometric evaluation of the connor-davidson resilience scale (CD-RISC) with chinese people. Soc. Behav. Pers. 35, 19–30. 10.2224/sbp.2007.35.1.19

[B68] Zhan N. Xie D. Zou J. Wang J. Geng F. (2021). The validity and reliability of benevolent childhood experiences scale in Chinese community adults. Eur. J. Psychotraumatol. 12, 1945747. 10.1080/20008198.2021.1945747 34290847 PMC8276668

[B69] Zheng X. Huang L. Xie Z. Peng L. Zhou X. (2022). Relationship between warm childhood memories and mobile phone addiction: a moderated mediation model. Psychol. Rep. 10.1177/00332941221135479 36269848

[B70] Zimmerman M. A. Stoddard S. A. Eisman A. B. Caldwell C. H. Aiyer S. M. Miller A. (2013). Adolescent resilience: promotive factors that inform prevention. Child Develop. Perspect. 7, 12042. 10.1111/cdep.12042 24288578 PMC3839856

